# Hedgehog signalling in the tumourigenesis and metastasis of osteosarcoma, and its potential value in the clinical therapy of osteosarcoma

**DOI:** 10.1038/s41419-018-0647-1

**Published:** 2018-06-13

**Authors:** Zhihong Yao, Lei Han, Yongbin Chen, Fei He, Bin Sun, Santosh kamar, Ya Zhang, Yihao Yang, Cao Wang, Zuozhang Yang

**Affiliations:** 1 Department of Orthopaedics, Bone and Soft Tissue Tumors Research Center of Yunnan Province, The Third Affiliated Hospital of Kunming Medical University (Tumor Hospital of Yunnan Province), Kunming, Yunnan 650118 China; 2 Key Laboratory of Animal Models and Human Disease Mechanisms, Kunming Institute of Zoology, Chinese Academy of Sciences, Kunming, Yunnan 650223 China; 3 Department of Orthopedics, The First Affiliated Hospital of Kunming Medical University, Kunming, Yunnan 650032 China

## Abstract

The Hedgehog (Hh) signalling pathway is involved in cell differentiation, growth and tissue polarity. This pathway is also involved in the progression and invasion of various human cancers. Osteosarcoma, a subtype of bone cancer, is commonly seen in children and adolescents. Typically, pulmonary osteosarcoma metastases are especially difficult to control. In the present paper, we summarise recent studies on the regulation of osteosarcoma progression and metastasis by downregulating Hh signalling. We also summarise the crosstalk between the Hh pathway and other cancer-related pathways in the tumourigenesis of various cancers. We further summarise and highlight the therapeutic value of potential inhibitors of Hh signalling in the clinical therapy of human cancers.

## Facts

- The Hh pathway regulates the progression of osteosarcoma.
- The Hh pathway is involved in the metastasis of osteosarcoma into other organs, such as the lungs.
- The Hh pathway crosstalks with other cancer-related pathways in the tumourigenesis of cancers.
- The therapeutic value of the Hh pathway in the clinical therapy of osteosarcoma is summarised.

## Open questions

- How does the Hh pathway regulate the tumourigenic progression and invasion of human osteosarcoma?
- How does the Hh pathway interact with other cancer-related pathways in the progression and metastasis of cancers?
- Could the Hh pathway be used as a target or biomarker in clinical therapy for human osteosarcoma?

## Introduction

Osteosarcoma, which is a malignant bone tumour with locally aggressive growth and high metastatic potential, is one of the most commonly observed diseases^1^. Distant metastases of osteosarcoma, such as lung metastases, are hard to control and usually have a poor prognosis^2^. The survival rate of osteosarcoma patients has gradually improved^3^. However, ~20% of osteosarcoma patients continue to present with lung metastases at diagnosis, and the 5-year survival rate has not significantly increased^4^. An exact description of the molecular basis of the proliferation and metastasis of osteosarcoma will help in the clinical treatment of osteosarcoma and improvement of patient survival.

Hedgehog (Hh)/Gli signalling is a conserved signal transduction pathway that possesses a key regulatory function in physiological processes, including embryonic development, tissue differentiation and cell growth^5,6^. Recently, the Hh pathway was found to possess a key function in the progression and metastasis of various cancers^7–10^. The Hh/Gli signalling pathway mainly includes the Hh ligand, its twelve-pass transmembrane protein receptor Patched (Ptc), the seven-pass transmembrane protein Smoothened (Smo), and cytoplasmic proteins involved in the Hh signalling protein complex, including Fused kinase, Costal-2 (Cos2), GSK3 beta, PKA, Fu suppressor protein (SuFu) and nuclear factor glioma-associated oncogene transcription factors, which are key downstream regulators in this signalling pathway and have a pivotal role in signal transduction^11–13^. Target genes in the Hh pathway are related to cell proliferation, survival, cell cycle, stem cell formation, cell invasion and many other processes^12^

In the present paper, we summarise the mechanism via which Hh/Gli signalling is regulated in the tumourigenesis and metastasis of cancers, focusing on the impact of these regulatory activities on the progression, invasion and metastasis of osteosarcoma. We also discuss the interaction between the Hh/Gli pathway and other cancer-related signalling pathways during the progression of human cancers. At the end of this review, we highlight the therapeutic value of Hh pathway inhibitors in the clinical therapy of human cancers, describe future challenges and propose possible directions for the Hh/Gli signalling-associated clinical treatment of osteosarcoma patients based on our current understanding.

## Introduction of Hedgehog signalling pathway

### Overview

**Fig. 1:**
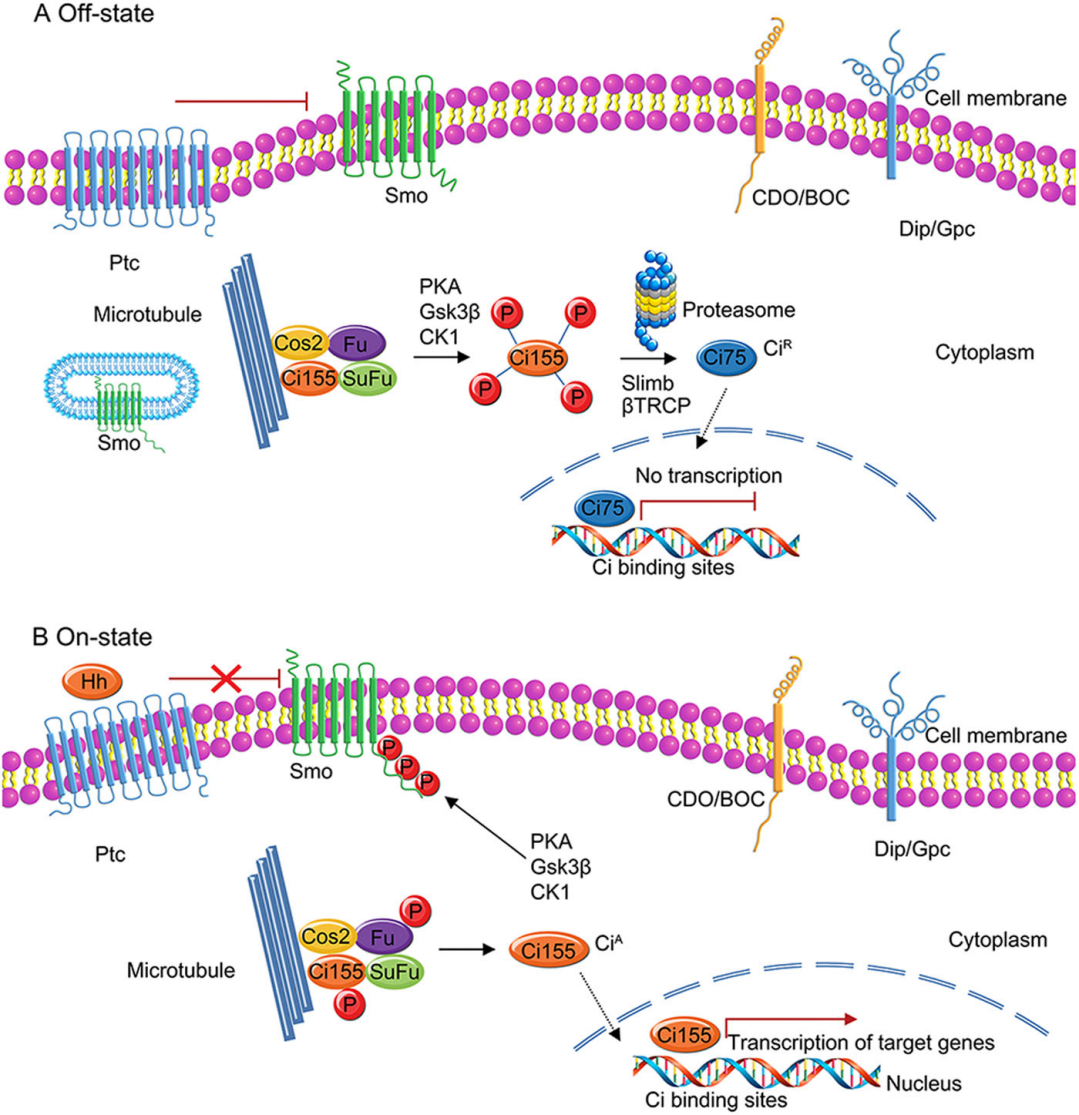
Schematic representation of the Hedgehog (Hh) signalling pathway in *Drosophila*. Briefly, in *Drosophila*, the core components of the Hh pathway include Hh ligands; the Patched receptor (Ptc); the transcription factor cubitus interruptus (Ci); the signalling activator G-protein-coupled receptor-like protein Smoothened (Smo); and a signal transducer, such as Costal (Cos2), Fused (Fu) or Suppressor of Fused (SuFu). **a** Off-state. The Hh signalling pathway is inactive in the absence of a Hh ligand. The seven-pass transmembrane protein Smo is repressed by Ptc. The target genes of the Hh signalling pathway are not transcribed or expressed. **b** On-state. In the presence of the Hh ligand, the Hh ligand binds to Ptc, and the repression of Smo is alleviated, initiating the classical Hh signalling pathway. The cascade of cytoplasmic protein complexes (such as Su, SuFu and Cos2) is activated, and the downstream transcription factor Ci translocates to the nucleus, leading to the transcription of target genes. Hh Hedgehog ligand, Smo Smoothened, PTCH1 Patched1, GLI glioma-associated oncogene family zinc finger

**Fig. 2:**
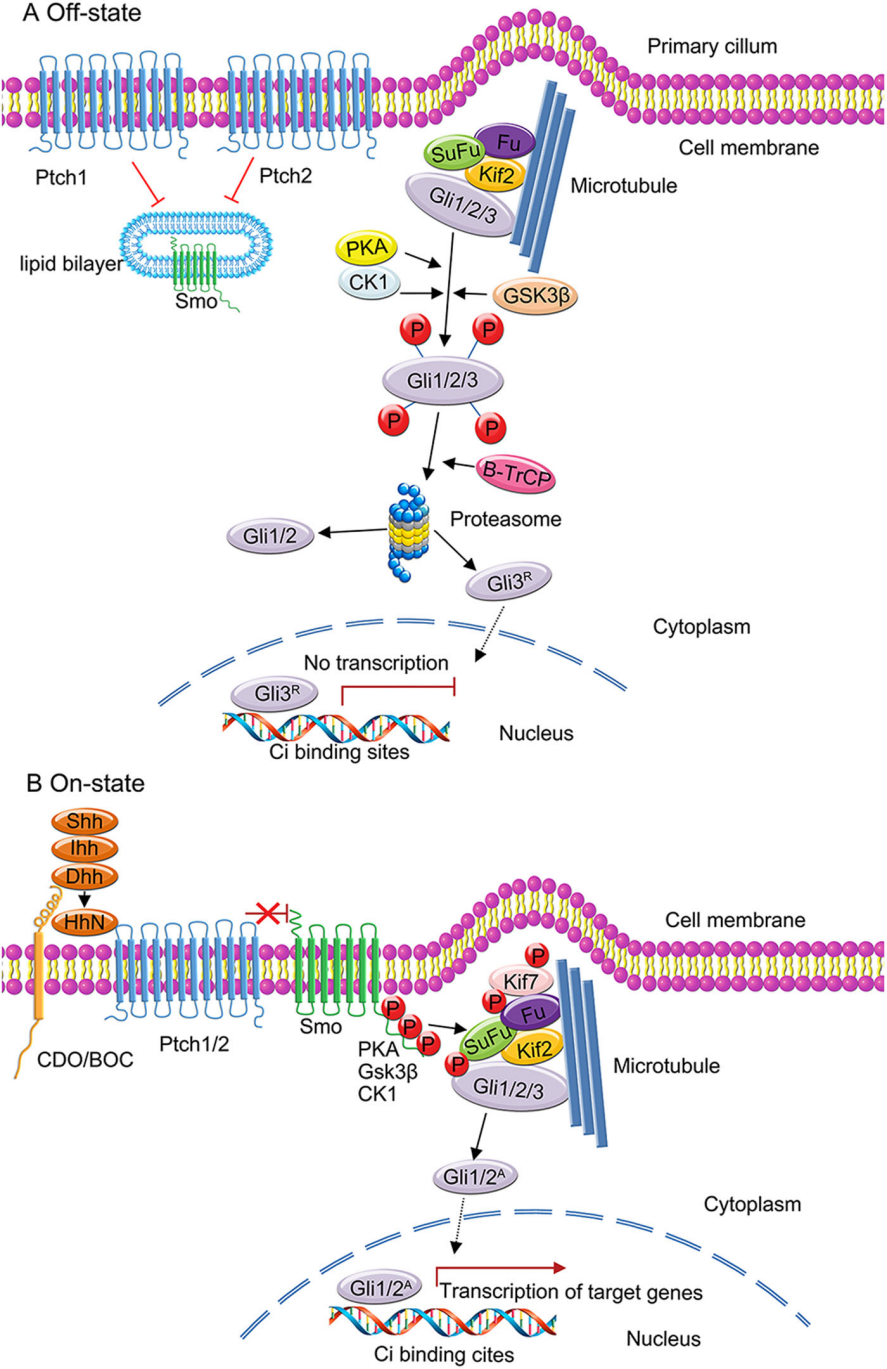
Schematic figure of the Hedgehog (Hh) pathway in vertebrates. In vertebrates, there are three Hh ligands, Sonic hedgehog (Shh), Desert hedgehog (Dhh) and Indian hedgehog (Ihh); two 12-pass transmembrane receptors, Patched1 (PTCH1) and Patched2 (PTCH2); Smoothened (Smo); and three transcription factor glioma-associated oncogene homologues, GLI1, GLI2 and GLI3. **a** Off-state. In the absence of the Hh ligands, the cell surface receptor Patched (Ptch1 or Ptch2) binds to the class F G-protein-coupled receptor Smo and inhibits Smo activity, resulting in the inhibition of Hh signalling pathway activation. **b** On-state. In the presence of a Hh ligand (Shh, Ihh or Dhh), the Hh ligand binds to its receptor Ptch1 or Ptch2, initiating the classical Hh signalling pathway. Hh induces the phosphorylation of multiple Ser/Thr residues at the carboxy terminus of Smo, resulting in the release of the inhibition of Smo by Ptch. The activated Smo recruits and activates a cascade of cytoplasmic protein complexes to activate the Hh pathway. The target genes of the Hh pathway are transcribed and expressed

Hh is a segmented polar gene that encodes a highly conserved secreted glycoprotein named for the bristly phenotype of the mutation of the gene in *Drosophila*. It was first identified in systematic searches for embryonic lethal mutants of *Drosophila melanogaster* by Nusslein-Volhard, C. in 1980^14^. The Hh/Gli pathway has a key regulatory function in physiological processes^15^. The Hh pathway is an important signalling pathway in the carcinogenesis and metastasis of several types of cancer^16–18^. This pathway is highly conserved and comprises some components that are regulated by post-translational events; however, there are some differences between *Drosophila* and higher organisms. Briefly, the core constituents of the Hh/Gli pathway in *Drosophila* are the Hh ligand; Ptc; cubitus interruptus (Ci); Smo; and signal transducers, such as Cos2, Fused (Fu), or SuFu(Fig. 1)^19–23^. In higher organisms, the core constituents of Hh signalling are more complex, comprising three Hh ligands, Sonic hedgehog (Shh), Desert hedgehog (Dhh) and Indian hedgehog (Ihh); two twelve-pass transmembrane receptors, Patched1 (PTCH1) and Patched2 (PTCH2); Smo; and three transcription factors, including GLI1, GLI2 and GLI3(Fig. 2)^24–27^. The three Gli nuclear transcription factors are highly similar in amino acid sequence. Usually, Gli1 is involved in transcriptional activation, while Gli2 and Gli3 can both activate and inhibit transcription^28–30^. Three homologous Hh ligands and Ptch can be combined and can perform different biological functions during different stages of cancer progression. The target genes of the Hh pathway involve different physiological processes, including stem cell formation and tumour cell invasion^31,32^ (Fig. 1).

Briefly, in the Hh pathway, in the absence of the Hh ligand, the cell surface receptor Ptc binds to Smo and effectively suppresses Smo activity, inhibiting the activation of Hh signalling^33,34^. At this point, Gli3 is phosphorylated and hydrolysed by proteolytic enzymes, releasing the transcriptional repressor protein Gli3 into the nuclear area and ultimately inhibiting the levels of the Hh target genes^35^. When the Hh ligand is present, it interacts with the receptor Ptch, initiating the classical Hh pathway. Hh induces the phosphorylation of multiple Ser/Thr residues at the carboxy terminus of Smo, resulting in the release of Smo inhibition by Ptch^28,36–38^. Smo is recruited and activated on the cell surface, which finally activates the cytoplasmic protein complex to activate the Hh signalling pathway^39^. The signal is transduced to Gli factor, which transports the signal to the cell nucleus and regulates the transcription of related genes involved in key cellular processes^40^.

### Regulation of the Hh pathway (miRNA, lncRNA, protein)

**Fig. 3:**
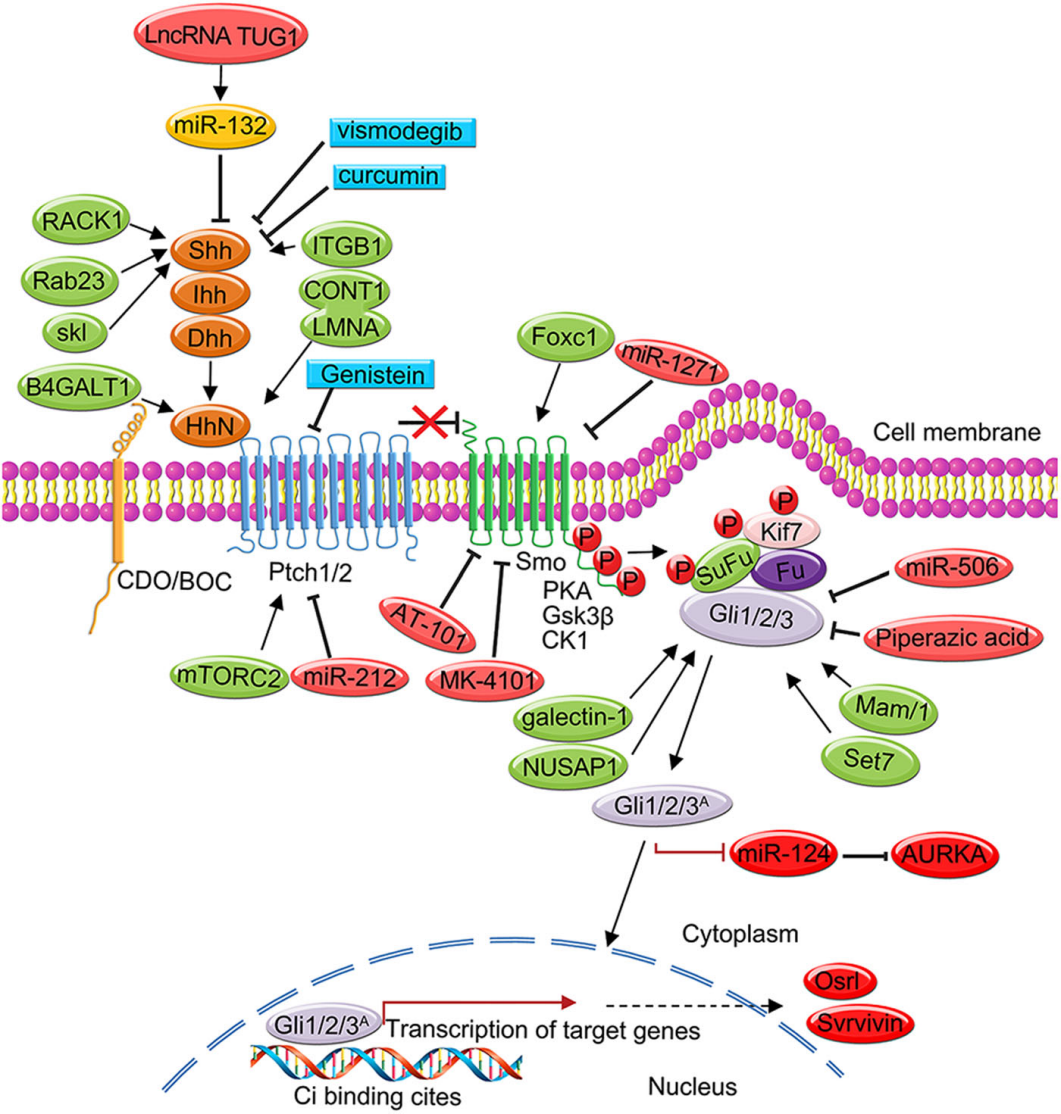
The Hedgehog (Hh) pathway is regulated by various factors, including proteins, lncRNAs, miRNAs, plant extracts and small molecules. The Hh pathway is constitutively active in various human cancers. Effective inhibitors of the Hh signalling pathway are used to suppress the inappropriate activation of the Hh pathway, which is beneficial and can be used for clinical therapies of human cancer. In this figure, we have summarised some factors, such as kinases, transcriptional factors, glycoproteins and proapoptotic or anti-apoptotic factors, that are involved in the abnormal activation of Hh signalling. Receptor of activated kinase 1 (RACK1), the Rab family of GTPases (Rab23), NIMA-related expressed kinase 2A (Nek2A), galectin-1 (Gal-1), beta1,4-galactosyltransferase 1 (beta1,4-Gal-T1), nucleolar and spindle-associated protein 1 (NUSAP1), Mastermind-like 1 (Maml1), Beta1 integrin (ITGB1), growth arrest-specific gene 1 (Gas1), Forkhead box C1 (FOXC1) and ribosomal protein S3 (RPS3)

Aberrant activation of the Hh pathway leads to the tumourigenesis and metastasis of human cancers, such as medulloblastoma, osteosarcoma, and gastric, lung, breast, colorectal and prostate cancers (Fig. 2). The Hh pathway controls tumourigenesis and tumour invasiveness in various cancers. An increasing number of agents effectively regulate signal transduction in the Hh signalling pathway; these agents include proteins, compounds, and noncoding RNA such as miRNA and lncRNA. Elucidation of the molecular basis of Hh signalling is beneficial and will help guide the clinical treatment of human cancers and screening of effective inhibitors to inhibit the inappropriate activation of the Hh pathway. Some of the important regulators in the Hh signalling pathway are summarised here and are shown in Fig. 3.

#### Positive and negative regulation of the Hh signalling pathway by various proteins

Some effective and key proteins, such as kinases, transcriptional factors, glycoproteins, and proapoptotic or anti-apoptotic factors, are accompanied by the abnormal activation of the Hh pathway in the progression of cancers (Fig. 3). Receptor of activated kinase 1 (RACK1) is overexpressed in non-small cell lung cancer, and the level of RACK1 is clearly associated with the pathological characteristics, such as tumour stage, differentiation and metastasis, of non-small cell lung cancer patients. Interference with RACK1 significantly suppresses cancer progression and metastasis because of the inhibition of the Shh pathway. RACK1 promotes the progression and metastasis of non-small cell lung cancers that primarily involve activation of the Shh pathway, in which RACK1 interacts with activated Smo to initiate Gli1 transcription in non-small cell lung cancer cells^41^. In hepatocellular carcinoma (HCC), inhibition of Rab23, a protein belonging to the Rab family of GTPases, negatively regulates the Shh signalling pathway and decreases the expression, nuclear translocation and localisation of Gli1 via GDP/GTP binding^42^. SuFu is highly conserved in the Hh pathway to impede the activation of downstream signalling. NIMA-related expressed kinase 2A (Nek2A) interacts with SuFu and stabilises SuFu. The Nek2A–SuFu complex inhibits the activation of Hh signalling and transcriptional activity of Gli2 by preventing the degradation of SuFu, which is mediated by the ubiquitin/proteasome system. Additionally, in a negative feedback loop, Gli1/Gli2 binds the promoter sequences of NEK2A and induces the transcription of NEK2A^43^. The K436 and K595 residues of full-length Gli3 can be methylated, which is catalysed by Set7. Methylation increases the stability and DNA-binding activity of Gli3 to promote Shh pathway activation. Moreover, Set7-mediated Gli3 methylation leads to cancer progression. Thus, Set7-mediated Gli3 methylation promotes Shh pathway activation in animals, and the Shh pathway promotes the tumourigenesis and metastasis of human cancers^44^. Galectin-1 (Gal-1) can be used as an indicator of poor survival in human gastric cancer patients. Another study demonstrated that Gal-1 levels are high and promote cancer metastasis and the ETM phenotype by activating the non-canonical Hh pathway. Specifically, this finding was indicated by an increase in the transcription of Gli1 via a Smo-independent pathway^45^. Beta1,4-galactosyltransferase 1 (beta1,4-Gal-T1) is encoded by the B4GALT1 gene. Beta1,4-Gal-T1 has an important function in disease progression by transferring galactose to acceptor sugars. Silencing B4GALT1 in K562/ADR cells using RNA interference suppresses the Hh signalling pathway, increases the sensitivity of human leukaemia K562/Adriamycin-resistant cells and reverses multidrug resistance^46^.

Smo mutations generally result in drug resistance in tumours. GTPase Arl13b is a newly identified partner and regulator of Smo, and Arl13b expression is closely associated with tumour size and invasion depth. Moreover, increased levels of Arl13b in patients often indicate a poor prognosis. Thus, Arl13b is involved in Hh pathway activation in human gastric cancer via the regulation of Smo stability, trafficking, and localisation^47^. Piperazic acid is a non-proteinogenic amino acid that downregulates Gli expression in the Hh signalling pathway and could be used as an effective tool to study Hh signalling^48^. Two types of Shh inhibitors, namely, vismodegib and sonidegib, are widely used. These drugs inhibit Smo, which significantly alleviates the progression of advanced BCC. However, these drugs often cause some side effects, such as muscle cramps, fatigue and weight loss^49,50^.

The transcriptional repressor proteins Gli1-3 are regulated by various proteins. For example, the level of NUSAP1 is clearly increased in several types of tumours. Overexpression of NUSAP1 induces Gli1 translocation into the nucleus in astrocytoma cells and upregulates a target gene of the Hh pathway, which is likely a valuable prognosis biomarker and a novel regulatory protein in astrocytoma^51^. Mastermind-like 1 (Maml1) binds Gli transcription factors as an effective transcriptional coactivator. Notably, Maml1 silencing leads to obvious downregulation of Gli target gene levels, suggesting that Maml1 cooperates with Gli factors to regulate the Shh pathway^52^. Beta1 integrin (ITGB1), the most highly expressed integrin protein, is normally overexpressed in human malignancies and contributes to tumourigenesis and metastasis. ITGB1 increases cell proliferation and invasion in colorectal cancer cells, which is mediated by the Hh pathway^53^.

#### The Hh/Gli signalling pathway is regulated by lncRNA and miRNAs

The lncRNA TUG1 has a role in cell proliferation, invasion and metastasis of cancers. Interference with TUG1 suppresses the proliferation of HCC via a miR-132- and Shh-related mechanism. TUG1 activates Hh signalling by competing with miR-132, which can directly bind to the 3′-UTR of Shh, leading to downregulation of miR-132 levels in HCC^54^. Thus, targeting the TUG1-miRNA132-Hh pathway could be a new strategy for the clinical treatment of human cancers. MiRNAs are also important regulators of the development and metastasis of various human cancers. miR-1271 directly targets Smo, suppresses cell growth and promotes the apoptosis of cell lines by suppressing the Smo-mediated Hh pathway^55^. Another report showed that miR-148a interacts with the 3′-UTR of growth arrest-specific gene 1(Gas1) and inhibits target gene expression. Overexpression of miR-148a increases autophagy and apoptosis to suppress the proliferation of hepatic stellate cells^56^. Additionally, The level of miR-212 is also increased in PDAC tissues and cells, and luciferase assays demonstrated that PTCH1 is the target of miR-212^57^. Together, these data suggest that miR-212 directly targets Ptch-1 and increases cancer cell proliferation and invasion. miR-506 suppresses the growth and viability of cervical cancer cells and in animal models. Elucidation of the molecular mechanism demonstrated that miR-506 promotes G1/S phase arrest, apoptosis and the chemosensitivity of human cervical cancer cells by directly targeting Gli3, revealing that the miR-506/Gli3 axis is a new potential therapeutic drug target for the clinical therapy of human cervical cancer^58^. miR-124 is a new downstream miRNA of Gli2. High levels of Gli2 inhibit the levels of miR-124, and elucidation of the molecular mechanism showed that Gli2 binds the upstream sequences of miR-124. Moreover, miR-124 directly interacts with the 3′-UTR of Aurka. High levels of Gli2 suppress the miR-124 level, leading to increased levels of AURKA in glioma cells. In human glioma cells, miR-124 is a key downstream target gene of the Hh pathway, and inhibiting the Hh pathway suppresses cell growth via the Gli2/miR-124/AURKA pathway^59^.

#### The Hh/Gli pathway in CSCs is regulated during tumourigenesis and metastasis

CSCs are involved in tumourigenesis and the invasion of human cancers. However, conventional treatments with anticancer drugs cannot eradicate CSCs, leading to the death of various cancer patients. Thus, targeting CSCs would be a novel and ideal method to cure human cancer. Osteosarcoma stem cells contribute to tumour recurrence, metastasis, and drug resistance via their self-renewal and differentiation^60^. The Forkhead box C1 (FOXC1) transcription factor promotes CSC properties in basal-like breast cancer, which could activate Smo-independent Hh signalling and Gli2, and provides novel insight into anti-Hh therapy resistance in cancer progression^61^. In human pancreatic CSCs, Ski has a key role in promoting tumour progression. In these cells, the Shh pathway is abnormally activated, and Ski enhances the levels of Shh pathway components, including Shh, Ptch1, Smo, Gli1 and Gli2. Additionally, depletion of Ski leads to the opposite effects^62^. Activated mTORC2 increases Gli2 stability and promotes nuclear translocation of Gli2 to regulate angiogenesis, invasion, cell proliferation and CSC regeneration^63^. Specifically, higher mTORC2 activity increases the levels of several Hh pathway molecules and promotes the mRNA and protein expression of target genes, which are closely correlated to increased metastasis, cell growth and cell differentiation. Moreover, elucidation of the molecular mechanism showed that mTORC2 suppresses Gli2 ubiquitination by inactivating GSK3 beta, thus increasing Gli2 stability and promoting nuclear translocation. Therefore, an mTORC2 inhibitor could be a new therapeutic for CSCs.

## Hh/Gli signalling and osteosarcoma

### Hh signalling in the local aggressive proliferation and distant metastasis of osteosarcoma

Osteosarcoma is a bone tumour in children with locally aggressive growth and high metastatic potential. Distant metastases of osteosarcoma, such as lung metastases, are typically hard to control and usually have a poor prognosis. Clarification of the mechanisms of tumourigenesis and metastasis of osteosarcoma will help in the clinical treatment of osteosarcoma and improvement of patient survival. Details regarding the mechanistic action of osteosarcoma, which include tumourigenesis, invasion and metastasis, have not been clearly elucidated until now. Osteosarcoma may be related to rapid bone growth in adolescence. Additionally, virus infection, ionising radiation and trauma may contribute to the occurrence of osteosarcoma^64–66^. In recent years, many new drugs for osteosarcoma have been developed globally with further clarification of the tumourigenesis of osteosarcoma, providing new means for clinical treatment. In the present article, we have summarised some important regulators associated with the Hh/Gli signalling pathway in osteosarcoma metastasis.

A target of Gli2 is the gene encoding ribosomal protein S3 (RPS3). Overexpression of RPS3 increases the metastasis of osteosarcoma cells. Importantly, immunohistochemical analysis demonstrated that RPS3 levels are much higher in osteosarcoma specimens with lung metastases than in those without metastasis. Thus, RPS3 regulates Hh/Gli2 signalling in the invasion and metastasis of osteosarcoma. RPS3 could be a molecular marker of invasive osteosarcoma and could likely be used as a therapeutic drug candidate for patients with aggressive and malignant osteosarcoma^67^. In another report, 210 osteosarcoma samples, 25 osteoblastomas and 19 osteosarcoma cell lines were examined, and the Smad-mediated and Gli-mediated signalling pathways were abnormally activated in advanced osteosarcoma^68^. Yes-associated protein 1 (Yap1) is an oncogene that is highly expressed in human osteosarcoma tissues. Inhibition of the Hh pathway decreases Yap1 levels, and the knockdown of Yap-1 clearly suppresses osteosarcoma progression. Moreover, the lncRNA H19 is abnormally expressed by the Hh/Gli signalling pathway and Yap1 overexpression, suggesting that the Hh pathway is an important regulator of osteoblasts during the tumourigenesis of osteoblastic osteosarcoma via the overexpression of Yap-1 and the lncRNA H19^69^. The small molecule IPI-926 (saridegib), a Smo antagonist, inhibits Hh signalling interactions in osteosarcoma, and Hh pathway inhibitors can be used as targeted therapeutics in the clinical treatment of osteosarcoma^70^. Degalactotigonin (DGT) is extracted from the plant *Solanum nigrum L*., and this molecule inhibits cell growth and migration and promotes programmed cell death, especially apoptosis in osteosarcoma cells. Importantly, DGT significantly decreased the rate at which osteosarcoma xenografts metastasised to the lungs and the volume of osteosarcoma xenografts. Elucidation of the molecular mechanism showed that DGT suppresses osteosarcoma proliferation and metastasis by inhibiting the Hh/Gli1 pathway, mainly via GSK3 beta inactivation^71^. MSCs are responsible for the formation of the tumour microenvironments and interactions with tumour cells. MSCs derived from human bone marrow (hBMSC)-derived exosomes increases osteosarcoma MG63 cell proliferation by activating the Hh pathway, while suppressing the Hh pathway obviously decreases tumour growth induced by hBMSC-derived exosomes^72^. CCR4-NOT transcription complex subunit 1 (CNOT1) was correlated with the cell growth of osteosarcoma cells via clinical screening and functional evaluation. CNOT1 interacts with lamin A and positively regulates the function of this intermediate filament protein. Moreover, the lamin A-dependent CNOT1 level was positively associated with the Enneking stage and tumour recurrence of patients with osteosarcoma by activation of the Hh/Gli pathway, and CNOT1 works as an oncogene in osteosarcoma development^73^. DeltaNp63 is a splice variant of p63, which is overexpressed in various human cancers and inhibits apoptosis. Elucidation of the function of DeltaNp63 in osteosarcoma shows that DeltaNp63alpha directly regulates the transcription factor Gli2. The functional interactions between DeltaNp63alpha and GLI2 promote the tumourigenesis of osteosarcoma cells^74^.

### Interactions between the Hh/Gli signalling pathway and other prominent cancer-related signalling pathways in malignant metastasis

Interactions between several signalling pathways, including the PI3K/AKT, ERK, Notch, Wnt and Shh pathways, control cell proliferation and metastasis in cancer progression. Different pathways may co-exist and interact with each other within a cell, ultimately functioning together to regulate cell metastasis. Understanding the interacting function between the Hh/Gli signalling pathway and other prominent pathways in cancer metastasis is helpful for the inhibition of cancer metastasis via combination therapy.

Microarray analysis data demonstrated that the “Hh pathway” and “Wnt pathway” are obviously upregulated. Moreover, IHH is overexpressed in metastatic cell lines compared with parental cell lines^75^, suggesting possible interplay between the Hh and Wnt pathways. The interaction between the Hh/Gli and PI3K/AKT pathways is an important factor that increases cancer metastasis during the tumourigenesis of osteosarcoma. One DNA microarray study demonstrated that 11 genes involved in the AKT/PI3K and Hh/Gli pathways are highly expressed in osteosarcoma (malignant) samples compared with osteoma (benign) samples, whereas the expression levels of 36 genes, such as HSPB8 and SEPP1, are downregulated^76^. Moreover, co-activation of both signalling pathways might be taken as a prognostic, diagnostic and recurrence marker for cancer patients. Another study revealed that activation of both pathways is closely related to poor outcomes of triple-negative breast cancer patients^77^. However, controversially, the authors revealed that suppression of the Wnt/β-catenin pathway and Notch pathway sensitises osteosarcoma cells to chemotherapeutic drugs. When used simultaneously, the inhibitors of the Wnt and Notch pathways show synergistic effects with the chemotherapeutic drug MTX, while treatment with the Hh inhibitors did not significantly increase drug efficiency^78^. The level of nuclear Gli1 is closely related to the pathological grade of tumours; invasive and metastatic abilities of the tumour; and levels of phosphorylated ERK1, phosphorylated ERK2 and MMP-9. Additionally, Shh and nuclear Gli1 are not detected in normal liver tissues. Importantly, U0126 and PD98059, which inhibit the MAPK pathway, suppress the invasive and metastatic abilities of liver cancer cells primed by Shh and downregulate the levels of p-ERK1/2 and MMP-9. Thus, during the metastasis of human HCC, the Hh pathway regulates the invasive abilities of human HCC cells by upregulating the MMP-9 levels via the ERK signalling pathway^79^. Hippo-YAP1 signalling is a cancer-related pathway that inhibits cell proliferation and metastasis. The major targets of the Hippo-YAP1 pathway are YAP and TAZ. Generally, the activities of YAP and TAZ are obviously suppressed via phosphorylation, which inhibits the translocation and accumulation of these proteins in the nucleus and leads to the inhibition of the co-transcriptional activity of these proteins. However, in cancer cells, an inactivated Hippo pathway results in free nuclear translocation of YAP and TAZ to regulate target genes involved in cell proliferation and metastasis. YAP activates the Shh pathway by increasing the levels of Ptch1. Thus, the Shh pathway acts downstream of YAP and affects cell proliferation and differentiation^80^. Tamoxifen-resistant breast cancer cells were treated with PI3K inhibitors, which downregulated the protein levels and activity of Smo and Gli1 and could be rescued by the inhibition of GSK3 beta and proteasomal degradation. These results suggest that Hh/Gli signalling is a novel and effective therapeutic target that is abnormally activated by the PI3K/AKT pathway^81^. Therefore, targeting the Hh pathway alone or combined with the PI3K/AKT pathway is an institutive method in the chemotherapeutic therapy of drug-resistant cancers. Additionally, cyclopamine inhibits Hh signalling and completely interrupts angiogenesis. In Hh-defective embryos, the levels and activities of the VEGF and Notch pathways are noticeably decreased, suggesting that Hh signalling is an important and substantial regulator of VEGF levels in the formation of new blood vessels^82^. Phosphatase and tensin homologue (Pten) is a PI3K pathway regulator. Hh pathway suppression significantly inhibits the proliferation of Pten-deficient medulloblastoma in an in vivo mouse experiment. Combined inhibition of the Hh and PI3K pathways results in more efficient antitumour effects in PTEN-deficient medulloblastomas^83^.

### Hh signalling and osteosarcoma prognosis and treatment

Osteosarcomas are highly malignant; however, with early diagnosis and appropriate treatment, osteosarcoma patients with a high-grade osteosarcoma at a single location have a survival rate of ~70%. The development of peripheral chondrosarcoma is characterised by downregulated levels of the Ihh signalling pathway, and parathyroid hormone-like hormone is independent of Ihh in peripheral chondrosarcomas^84^. Ihh is a cytokine produced by osteoblastic cells. The post-transcriptional activity of lhh is involved in and regulated by TGF-β^85^. However, higher expression of the Hh ligand (Ihh) and its downstream target genes PTCH1 and Gli1 was observed in 43 clinical specimens of high-grade human osteosarcoma. Further analysis showed both the Ihh and Ptch1 genes are expressed in large tumours, and higher expression of Ihh is prevalent in men. Patients who exhibit high expression of Gli1 are more responsive to chemotherapeutic drugs. Thus, higher levels of Hh signalling are associated with high-grade human osteosarcoma progression^86^. However, another report found that higher levels of Ihh are not associated with any clinicopathological parameter, as determined by immunohistochemical staining of Ihh in 48 tissue microarrays. Thus, Ihh is not an appropriate prognostic marker of osteosarcoma^87^. An analysis of 206 specimens of bone tumours showed that among the indicators with diagnostic utility, including SP7, IHH, RUNX2, SOX9 and TWIST1, only RUNX2, TWIST1 and SOX9 are sensitive and specific factors that differentiate between chondroblastoma and chondromyxoid fibroma. In contrast, Ihh did not have added value for the diagnosis and prognosis of this disease^88^.

### Potential tumour suppressor drugs targeting the Hh pathway

**Fig. 4:**
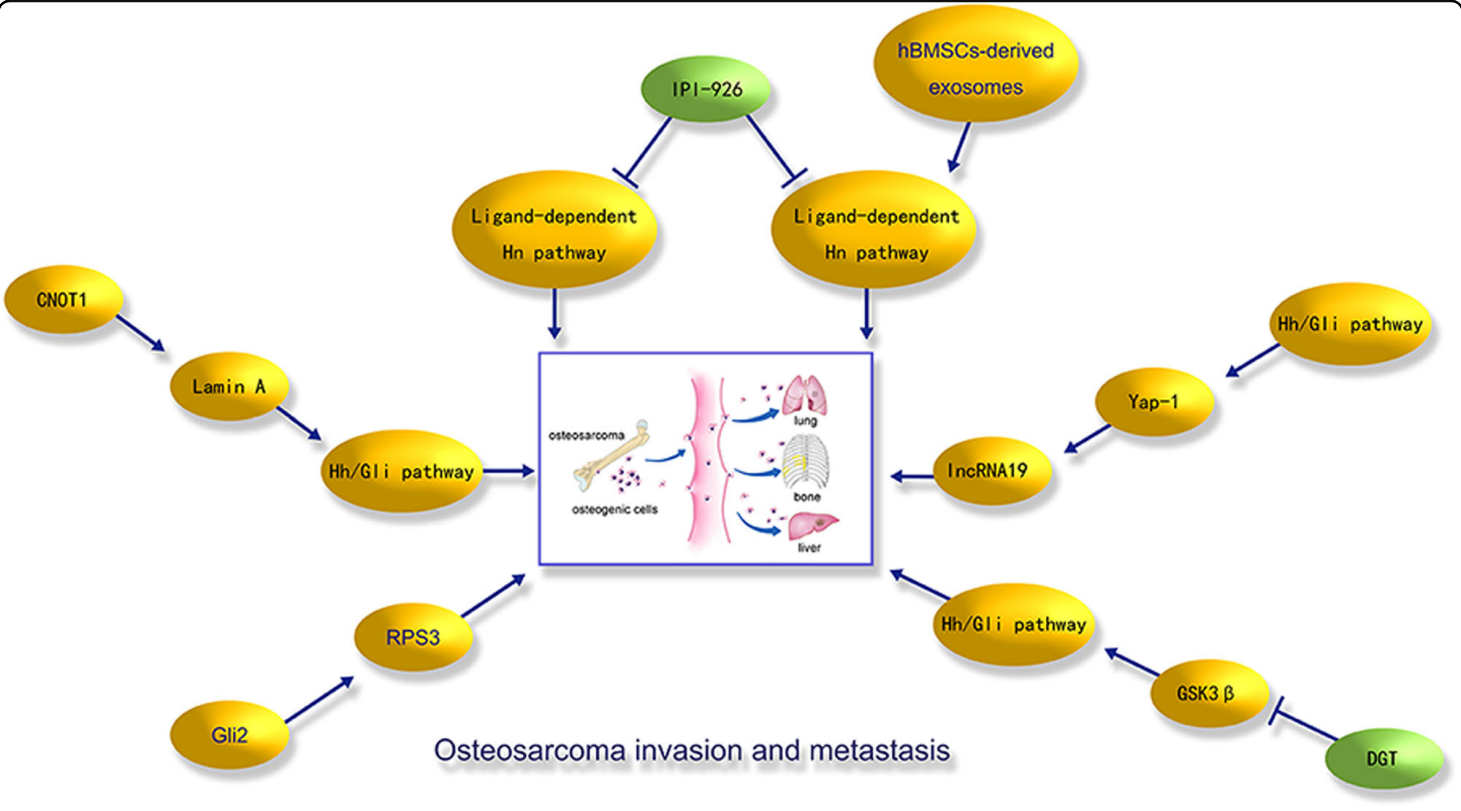
Hedgehog (Hh) signalling in the local aggressive proliferation and distant metastasis of osteosarcoma. Osteosarcoma is a malignant bone tumour with locally aggressive growth and high metastatic potential. Distant metastases of osteosarcoma, such as lung, bone and liver metastases, are frequently observed. Some important regulators that are associated with the Hh/Gli signalling pathway in the invasion and metastasis of osteosarcoma have been summarised

Osteosarcoma patients are normally cured and treated by surgery or chemotherapy, but most patients relapse because of distant metastasis to other organs after the initial treatment^89,90^. Thus, it is imperative to explore and identify effective targets and new clinical therapeutic strategies. Abnormal Hh signalling promotes metastatic ability and the progression of osteoblastic osteosarcoma (Fig. 4). Hh/Gli pathway inhibitors prevent osteosarcomagenesis, and inhibiting Hh signalling increases the efficacy of osteosarcoma therapy and improves patient outcomes^91,92^. The Shh signalling pathway is involved in the radioresistance of human osteosarcoma cells, and emodin demonstrates effective inhibitory effects on the radioresistance of osteosarcoma cells by suppressing the Shh pathway^93^. The Hh pathway is dysregulated during the development of osteosarcoma, and five novel potent Smo inhibitors inhibit osteosarcoma cell proliferation and obviously reduce Gli1 protein levels, suggesting that inactivation of Smo could be an effective approach in the clinical therapy of patients with osteosarcoma^94^. Gli2 is overexpressed in several osteosarcoma cells, and its overexpression is related to poor clinical results in osteosarcoma patients. Importantly, interference of Gli2 inhibits osteosarcoma cell growth^95^. Moreover, the antitumour drug arsenic trioxide (ATO) decreases the invasive ability of osteosarcoma cells by suppressing the transcriptional activity of Gli2^96^. Additionally, ATO prevents osteosarcoma development by inhibiting Gli transcription via the accumulation of DNA damage^97^. The Gli-specific inhibitor GANT61 suppresses the levels of Gli1/2, Ptch1 and Pax6, cell growth and colony formation in osteosarcoma cells^98^. LDE225 is a selective Smo antagonist and Hh signalling inhibitor that might be a promising anticancer drug. Osteosarcoma-bearing mice were orally administered with LDE225, and in vivo tumour growth was clearly suppressed in an immunocompromised setting, suggesting that LDE225 works mainly via a cytostatic effect but does not regulate the immunogenicity of tumour cells^99^. Cyclopamine, a steroidal alkaloid and Hh inhibitor, is highly effective against osteosarcoma cells, including HOS, SaOS and OS-KA, and significantly suppresses the proliferation and induces the death of osteosarcoma cells^100^. Cyclopamine suppresses the Hh pathway by binding to Smo. In an animal model of pulmonary metastasis, the cyclopamine group showed a trend of ~20% inhibition of the metastasis, suggesting that cyclopamine has obvious inhibitory effects on pulmonary osteosarcoma metastasis in vivo; however, marked side effects, mainly skin ulcerations, are caused by cyclopamine/ethanol/triolein administration^101^. High-dose chemotherapy and surgical intervention are common choices for the clinical treatment of osteosarcoma and have improved the prognosis for osteosarcoma patients without metastasis to 50–80% Table 1. However, metastatic osteosarcoma is resistant to common chemotherapies and remains an obstacle that affects clinical outcomes. Combinations of Hh inhibitors, including ATO and vismodegib, with anticancer drugs have synergistic antitumour effects when the compounds are used to treat human osteosarcoma cells (143B and Saos2), and this method may be a novel therapeutic therapy in the treatment of osteosarcoma^102^.

**Table 1:** Potential tumour-suppressing drugs targeting the Hedgehog pathway in osteosarcoma

Agent	Target	Description
Gli2 siRNA	Gli2	Interference of Gli2 by siRNA downregulates cell viability and upregulates the sensitivity of osteosarcoma cells to chemotherapeutic drugs^95,96^.
LDE225	Smoothened antagonist	In an osteosarcoma-bearing murine model, the antitumor activity mainly involved a cytostatic effect after oral administration of LDE225^99^.
Cyclopamine	Steroidal alkaloid and Hh inhibitor targeting Smoothened	Cyclopamine significantly inhibits cell proliferation and induces cell death in osteosarcoma cell lines and inhibits pulmonary metastasis in murine models^100,101^.
Arsenic trioxide (ATO), Vismodegib, GANT61	Hh inhibitor	Combined treatment with Hh pathway inhibitors, such as ATO and vismodegib, and standard FDA-approved anticancer agents shows synergistic antitumor effects on osteosarcoma cells^102^. Moreover, a combination of Hh inhibitors suppressed the invasion and metastasis of osteosarcoma cells^96,98^.
Genistein	Hedgehog-Gli1 pathway inhibitor	Genistein is a major isoflavone constituent extracted from traditional plant-based sources, such as soybeans and soy products. Genistein suppressed the stemness of cancer cells via the Hh/Gli1 pathway^103^.
Curcumin	Shh signalling inhibitor	Curcumin, a major component of phytochemicals, is able to promote the cell apoptosis of MG63 cells via the mitochondrial pathway^104^ and also suppresses the Shh activation in CSCs^105^.

## Conclusion and perspectives

Previous studies found that aberrant Hh/Gli pathway signalling promotes tumourigenesis and the aggressiveness or metastasis of tumours. Exploring effective Hh/Gli signalling inhibitors could provide novel and exciting clinical treatment methods for various cancers. In the present article, we discussed the role and function of the Hh pathway in the cell proliferation, invasion and metastasis of osteosarcoma and the crosstalk between the Hh pathway and other key cancer-related pathways, including the PI3K/AKT, ERK, Notch and Wnt signalling pathways. Moreover, the therapeutic role of the Hh pathway in the clinical therapy of osteosarcoma was also summarised. However, a few important points require further exploration.

1. Although several inhibitors of the Hh/Gli pathway, including ATO, have been thoroughly investigated and effectively inhibit the invasion and metastasis of osteosarcoma cells by inhibiting Gli2 activities^96^, some side effects remain in the clinical treatment of human cancers. More effective Hh/Gli signalling inhibitors with fewer side effects are necessary to inhibit the tumourigenesis, invasion and metastasis of osteosarcoma.
2. Because Hh/Gli inhibitors represent a novel and promising strategy in the prognosis and clinical treatment of osteosarcoma, the potential clinical applications of effective inhibitors need to be further explored.
3. An increasing number of regulators and target genes of the Hh/Gli pathway is gradually being identified. However, the molecular mechanism of the Hh/Gli pathway in the tumourigenesis and metastasis of osteosarcoma is not completely understood. Programmed cell death, including apoptosis, autophagy and necroptosis, has a key role in the progression and invasion of osteosarcoma. Whether and how the Hh/Gli signalling pathway regulates programmed cell death in the invasion and metastasis of osteosarcoma to distant organs remain to be explored.
